# Complementary efficacy of CD127‐directed immunotherapy with lusvertikimab and ABL‐targeting tyrosine kinase inhibitors in preclinical ABL‐class‐fusion‐positive B‐ALL

**DOI:** 10.1002/hem3.70407

**Published:** 2026-06-17

**Authors:** Denis Pinkle, Melissa Cornils, Anna Dietterle, Anna Laqua, Michelle Kurschies, Julia Heymann, Emily Funke, Philipp Fanter, Dennis D. Gupta, Claas Reimer, Denny Takudzwa Mahachi, Carina L. Gehlert, Dorothee Winterberg, Michaela Vossen‐Gajcy, Xenia Müller, Greta Balow, Katharina Iben, Malwine Barz, Lorenz Bastian, Claudia Baldus, Maja Kowol, Sally Kikvadze, Alexander A. Wurm, Ekaterina Logvinova, Christian Vokuhl, Jan P. Schmid, Irmela Jeremias, Katja Klausz, Matthias Peipp, Martin Schrappe, Irène Baccelli, Nicolas Poirier, Monika Brüggemann, Gunnar Cario, Denis M. Schewe, Lennart Lenk

**Affiliations:** ^1^ Department of Pediatrics I, ALL‐BFM Study Group Christian‐Albrechts University Kiel and University Medical Center Schleswig‐Holstein Kiel Germany; ^2^ University Cancer Center Schleswig‐Holstein (UCCSH) University Medical Center Schleswig‐Holstein Kiel Germany; ^3^ Medical Department II Hematology and Oncology University Medical Center Schleswig‐Holstein Kiel Germany; ^4^ Clinical Research Unit CATCH ALL KFO Kiel Germany; ^5^ Department of Pediatric Hematology and Oncology University Hospital Dresden Dresden Germany; ^6^ National Center for Tumor Diseases (NCT/UCC) Dresden Dresden Germany; ^7^ German Cancer Consortium (DKTK) Partner Site Dresden Dresden Germany; ^8^ Section of Pediatric Pathology, Department of Pathology University Hospital Bonn Bonn Germany; ^9^ Research Unit Apoptosis in Hematopoietic Stem Cells Helmholtz Center Munich Munich Germany; ^10^ OSE Immunotherapeutics Nantes France

Immunotherapy, especially with CD19‐targeting agents, has become a cornerstone of B‐cell precursor acute lymphoblastic (B‐ALL) therapy. Yet, not all patients benefit from these approaches, as immune escape may limit treatment success, and relapses frequently occur, particularly in some high‐risk subgroups.^1^

One important high‐risk subgroup is B‐ALL bearing ABL‐class‐fusions. This includes *BCR::ABL1‐*positive and *BCR::ABL1‐*like B‐ALL with related rearrangements involving *ABL1, ABL2, CSF1R, LYN, PDGFRA*, and *PDGFRB*,^2^ here collectively termed ABL‐class‐fusion‐positive (+) ALL.

The addition of tyrosine kinase inhibitors (TKIs) such as imatinib and dasatinib to chemotherapy backbones has markedly improved outcomes by directly targeting the aberrant kinase activity. Yet, resistance to TKIs, severe treatment‐related toxicity, and relapsed/refractory disease (r/r) remain significant clinical challenges, illustrating the need for new therapy options like immunotherapy in ABL‐class‐fusion+ B‐ALL.^2^

A promising target is the interleukin‐7‐receptor‐α (IL‐7R, CD127), which, upon binding with IL‐7, forms a heterodimer with the common gamma‐chain, triggering downstream signaling through JAKs and STAT5.^3^ CD127 signaling is important for the development of normal and malignant B‐ and T‐cells in mice and humans, with a non‐redundant role demonstrated for a *BCR::ABL1* mouse model.^4^, ^5^, ^6^, ^7^

Recently, we showed preclinical efficacy of CD127‐directed immunotherapy (CD127‐IT) with the monoclonal antibody lusvertikimab alone and in combination with chemotherapy in a comprehensive cohort of B‐ and T‐cell (T‐)ALL patient‐derived xenograft (PDX)‐samples. CD127 surface expression on PDX‐cells was identified as a predictive biomarker for lusvertikimab efficacy.^8^ Of note, lusvertikimab exposed a favorable safety profile in a phase‐1 study with 63 healthy volunteers and clinical efficacy in a phase‐2 trial of Ulcerative colitis (NCT04882007) without higher‐grade lymphopenia or T‐cell compartment alterations.^9^

Based on this rationale, we aimed to investigate the preclinical efficacy of lusvertikimab in ABL‐class‐fusion+ ALL, especially in the context of TKI treatment.

First, to determine the number of pediatric ABL‐class‐fusion+ patients that may potentially benefit from CD127‐IT, CD127 surface expression was prospectively measured via flow cytometry in diagnostic B‐ and T‐ALL samples in accordance with EuroFlow and iBFM Flow guidelines.^8^, ^10^, ^11^ For this, we extended our previously published analysis to 855 ALL patients,^8^ of which 44 (5.1%) were ABL‐class‐fusion+ (25 *BCR::ABL1*+ (2.9%) and 19 *BCR::ABL1*‐like (2.2%)).

We detected CD127‐positivity (CD127^pos^, defined as >10% CD127^+^ blasts) in 79.1% of patient samples‐ 40.3% of cases were highly positive for CD127 (CD127^high^, defined as >50% CD127^+^ blasts, Figure 1A). Among ABL‐class‐fusion+ cases, 56.0% of *BCR::ABL1*+ and 68.4% of *BCR::ABL1*‐like cases were CD127^pos^, 20.0% and 21.0% of cases were CD127^high^, respectively (Figure 1A).

**Figure 1:**
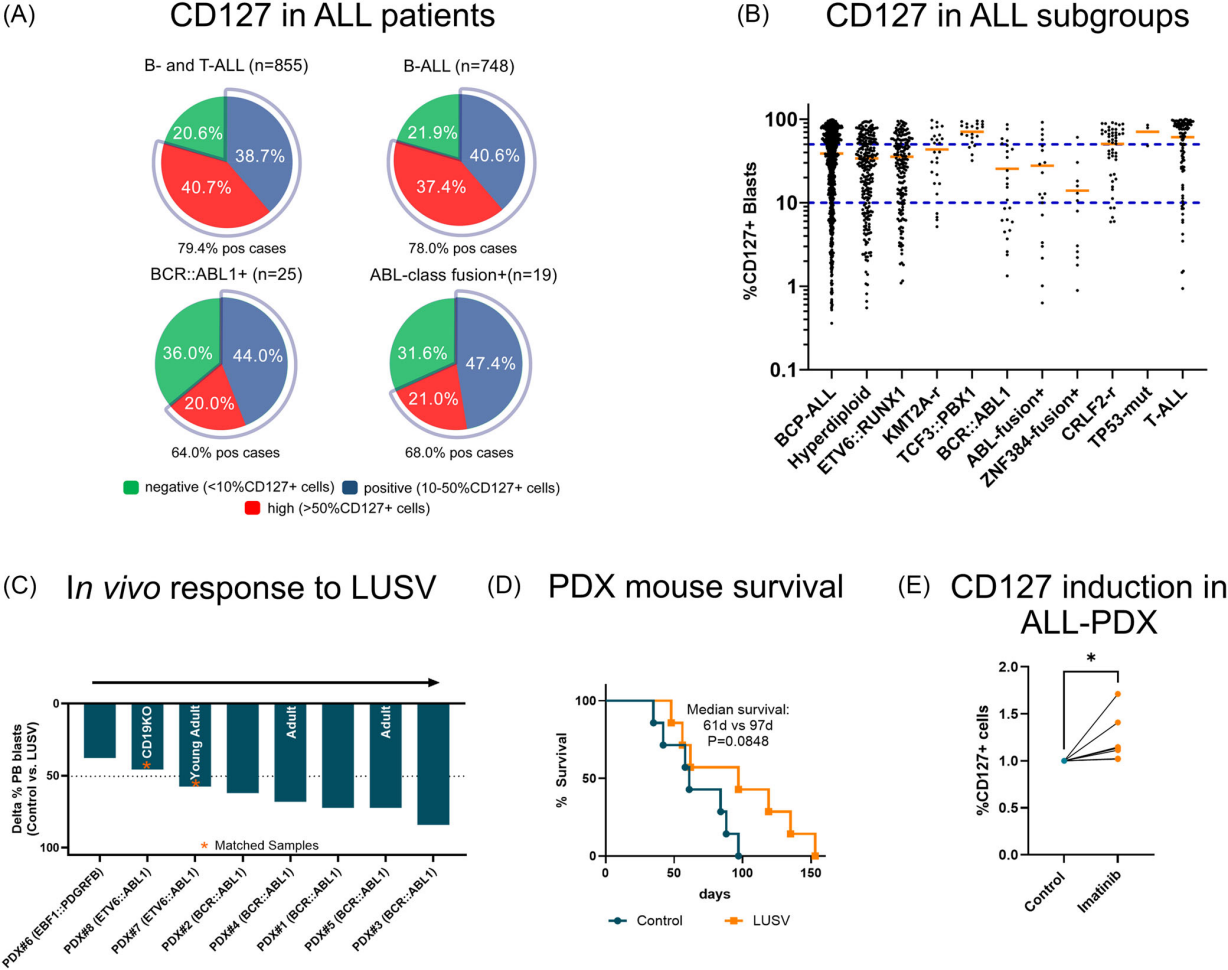
**CD127 is targetable in ABL‐class‐fusion‐positive ALL and its expression can be enhanced by imatinib treatment**. **(A, B)** CD127 surface expression was prospectively measured via flow cytometry in 855 diagnostic blood or bone marrow samples of B‐ALL and T‐ALL patients in accordance with International‐BFM‐FLOW recommendations.^11^ CD127 low positivity was defined as ≥10% CD127^+^ ALL cells by flow cytometry and high expression as ≥50% CD127^+^ blasts within the CD45^dim^/CD19^+^ (B‐ALL) or CD45^dim^/CD7^+^ (T‐ALL) cell population, respectively. **(A)** Pie charts depicting the ratio of CD127‐negative, CD127‐low, and CD127‐high ALL patients among all analyzed patient samples (top left), B‐ALL (top right), *BCR::ABL1*+ (bottom left) or *BCR::ABL1‐like ABL‐class‐fusion‐positive* (bottom right) cases within the cohort. **(B)** Ratio of CD127^+^ ALL cells in different subgroups. The stratification relevant lesions *ETV6::RUNX1*, *BCR::ABL1*, *TCF3::PBX1,* and *KMT2A*‐rearrangements (*KMT2A*‐r) were diagnosed by fluorescence in situ hybridization or RNA‐sequencing. Further genetic alterations (*CRLF2*‐rearrangements (*CRLF2*‐r), *TP53*‐mutations (*TP53*‐mut), and *ZNF384*‐fusions (*ZNF384*) were detected within the “B‐others” subgroup (B‐ALL cases without *ETV6::RUNX1, BCR::ABL1, TCF3::PBX1,* and *KMT2A‐r*). Blue lines show the cutoffs for low and high CD127‐positivity. **(C, D)** A phase‐2‐like PDX study was performed using *n* = 8 PDX samples from different ABL‐class‐fusion+ patients (PDX1‐8, Supporting Information S1: Table 1). Two NSG‐mice per patient were injected with PDX‐cells, randomly assigned into treatment groups and lusvertikimab (LUSV)‐therapy was initiated upon detection of 1% PDX‐cells in the peripheral blood (PB), modelling an overt leukemia situation. **(C)** Blood of both control and lusvertikimab‐treated animals bearing the same PDX sample was withdrawn when one of the two PDX mice showed signs of overt leukemia, and the number of hCD45+/hCD19+/mCD45− cells in the PB was measured via flow cytometry. The waterfall plot shows the difference in PB blasts between respective control and lusvertikimab‐treated mice (Delta PBB, sorted from weakest therapy response to highest therapy response). Animals not showing clinical signs of overt leukemia or >70% PB‐blasts at this timepoint received further treatment until reaching termination criteria. The dotted line indicates a delta PBB of 50%. The yellow asterisk indicates matched samples from the same patient, representing a control sample (PDX#7) and a CD19‐negative sample generated from PDX#7 by CRISPR‐Cas9 editing (PDX#8). **(D)** Therapy‐associated differences in the survival of NSG mice was determined using Kaplan‐Meier log‐rank statistics. The CD19‐negative PDX#8 was considered a proof‐of‐concept sample for CD19‐negative ALL and not included in Kaplan–Meier log‐rank analysis. **(E)** ABL‐class‐fusion+ PDX cells were exposed to sublethal doses of imatinib (Ima, 1 µM) or DMSO control and subjected to CD127‐assessment via flow cytometry after 48 h. A two‐tailed Wilcoxon matched‐pairs signed rank test of *n* = 7 PDX samples is shown. *P < 0.05. DMSO, dimethyl sulfoxide.

In line with previous results, the comparative analysis of ALL subgroups exposed a lower frequency of CD127^pos^ cases in ABL‐class‐fusion+ ALL than in most other B‐ALL subgroups (median CD127^+^ cells 26.44, 95% confidence interval (CI) [18.49, 34.39] vs. 37.46, [35.33, 39.94], P = 0.0029, Figure 1A,B).^6^

Overall, these data show that a substantial proportion of ALL cases are CD127‐positive and could potentially benefit from CD127‐IT, including relevant subsets of *BCR::ABL1*‐positive and ABL‐class‐fusion+ *BCR::ABL1*‐like cases.

Next, we tested the efficacy of lusvertikimab monotherapy in vivo in ABL‐class‐fusion+ PDX‐models using a preclinical phase‐2‐like PDX‐study design (Supporting Information S1: Figure 1A).^12^ The PDX‐cohort comprised 8 samples derived from 7 individual patients: 3 pediatric and 2 adult *BCR::ABL1*+ (PDX#1‐5), 1 pediatric *EBF1::PDGFRB* + (PDX#6), and 1 young adult r/r *ETV6::ABL1*+ (PDX#7) PDX‐samples (Supporting Information S1: Table 1). From PDX#7, we also included a CD19‐negative derivative generated by CRISPR/Cas9 editing as a proof‐of‐concept model for CD19‐negative relapse (PDX#8, Supporting Information S1: Figure 1B).^13^

Two mice per patient were randomly assigned to treatment groups, and therapy was initiated when mice showed 1% blasts in the peripheral blood (PB), mimicking an overt leukemia situation^14^, ^15^ (Supporting Information S1: Figure 1C). When one of the two mice (control or lusvertikimab therapy) developed clinical signs of leukemia, the peripheral blood of both PDX‐mice was analyzed for B‐ALL cells.^8^, ^14^ A blast reduction was observed in 8/8 (100%) of lusvertikimab‐treated PDX‐animals (37.6%–84.0% delta PB blasts, Figure 1C). A prolongation of survival in lusvertikimab‐treated animals as compared to the control group was also observed (P = 0.085, Figure 1D). The blast reduction between PDX#7 and PDX#8 was comparable, indicating lusvertikimab efficacy also upon CD19‐loss (Figure 1C). Of note, the magnitude of in vivo response to lusvertikimab correlated with the frequency of CD127‐positive cells as determined by flow cytometry (Pearson *r* = 0.7603; P = 0.047, Supporting Information S1: Figure 1D).

We had previously shown that treatment with noncytotoxic imatinib doses enhances CD127 expression in a murine *BCR::ABL1*+ preB‐cell model.^6^ Since lusvertikimab efficacy correlates with CD127 expression levels,^8^ we next tested if expression of CD127 can be enhanced by imatinib in ABL‐class‐fusion+ ALL cells. Similar to the murine pre‐B‐cell model, exposure to noncytotoxic doses of imatinib (1 µM) significantly enhanced CD127 expression in the *BCR::ABL1*+ cell line SUP‐B15 and in a variety of ABL‐class‐fusion+ PDX‐models, particularly in CD127^low^ samples (P = 0.0286 and P = 0.0156, respectively; Figure 1E, Supporting Information S1: Figure 2A–D), substantiating the plasticity of CD127 expression in ABL‐class‐fusion+ ALL cells in response to TKI.

Lusvertikimab is an IgG4 antibody which mainly acts via induction of antibody‐dependent cellular phagocytosis (ADCP) through binding of CD64 on macrophages and via pathway blockade.^3^, ^8^ Hence, we tested the effect of lusvertikimab and imatinib on macrophage‐mediated phagocytosis of ABL‐class‐fusion+ ALL cells employing macrophages from healthy human donors as effector cells. Both imatinib and lusvertikimab significantly enhanced macrophage‐mediated phagocytosis in SUP‐B15 cells as monotherapies (P = 0.0022 and P = 0.0087, respectively). The strongest in vitro phagocytosis induction was observed when imatinib and lusvertikimab were combined (P = 0.002, Supporting Information S1: Figure 3A). Of note, comparable effects were observed for ABL‐class‐fusion+ ALL‐PDXs showing enhanced ADCP upon imatinib/lusvertikimab combination in 8/8 tested samples (P = 0.0002, Figure 2A, Supporting Information S1: Figure 3B). This shows that imatinib enhances the ADCP‐promoting capacity of lusvertikimab in ABL‐class‐fusion+ ALL.

**Figure 2:**
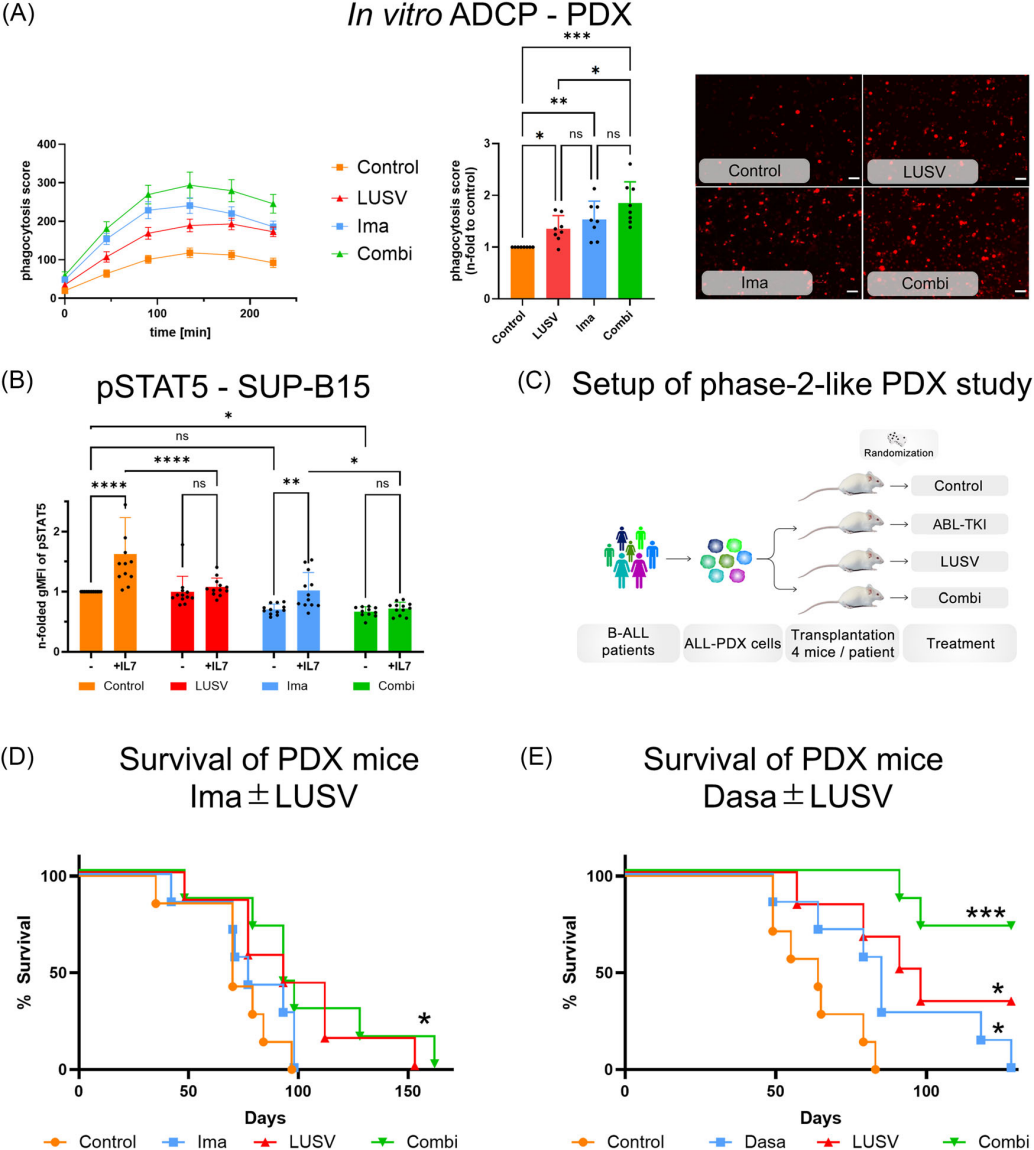
**ABL‐targeted TKI enhance the in vitro and in vivo efficacy of lusvertikimab in ABL‐class‐fusion‐positive PDX‐ALL**. **(A, B)** ABL‐class‐fusion+ patient‐derived xenograft (PDX) ALL cells were treated with sublethal doses of imatinib (Ima, 1 µM) for 48 h versus DMSO control and analyzed for phagocytosis/ADCP in the presence or absence of lusvertikimab (LUSV) using primary macrophages from healthy donors as effector cells and IncuCyte live‐cell imaging as readout. **(A)** Phagocytosis levels of a representative experiment with ALL‐PDX cells (one‐way ANOVA of *n *= 8 individual PDX samples) and representative images of pHrodo‐stained ALL cells are shown. Phagocytosed cells are stained in red, the white bar represents a scale of 50 µm. **(B)** The effect of STAT5‐phosphorylation (pSTAT5) in BCR::ABL1+ SUP‐B15 cells treated with imatinib and/or lusvertikimab in the presence or absence of recombinant IL‐7 was analyzed. Analysis of *n *= 6 individual experiments (two‐way ANOVA) are shown. **(C–E)** A phase‐2‐like PDX study was performed using 8 PDX samples of different ABL‐class‐fusion+ patients. Four NSG mice per patient were injected with PDX cells, randomly assigned into treatment groups, and lusvertikimab and/or ABL‐TKI therapy was initiated upon detection of 1% PDX cells in the PB, modeling an overt leukemia situation. **(C)** Schematic depiction of the experimental design. Therapy‐associated differences in the survival of NSG mice treated with **(D)** imatinib, lusvertikimab, or both (combi) and **(E)** dasatinib (Dasa), lusvertikimab, or the combination of both were determined using Kaplan–Meier log‐rank statistics. The CD19‐negative PDX#8 (generated from PDX#7 by CRISPR‐Cas9 editing) was considered a proof‐of‐concept sample for CD19‐neg ALL and not included in Kaplan–Meier log‐rank analyses depicted in panels D and E. *P < 0.05; **P < 0.01; ***P < 0.001; ****P < 0.0001. DMSO, dimethyl sulfoxide.

Exogenous IL‐7 can counter the antileukemic effect of imatinib in *BCR::ABL1*‐transformed pre‐B‐cells, partly by upregulation of the IL‐7R‐pathway.^6^ Apart from ADCP induction, lusvertikimab may exert direct IL‐7R‐pathway blocking effects, resulting in reduced phospho‐(p)STAT5 and cell viability.^3^, ^8^ Hence, we next tested if simultaneous blockade of intrinsic and extrinsic IL‐7R‐signaling by imatinib and lusvertikimab may effectively target ABL‐class‐fusion+ ALL cells. In line with previous results, we observed a significant pSTAT5 induction upon IL‐7‐stimulation in SUP‐B15 cells, indicating extrinsic pathway activation (P < 0.0001, Figure 2B). Treatment with imatinib alone reduced basal pSTAT5. However, exogenous IL‐7 restored pSTAT5 to baseline levels, indicating an externally mediated compensation of IL‐7R‐pathway activity. Lusvertikimab monotherapy abrogated IL‐7‐mediated STAT5 activation (P < 0.0001), suggesting effective blockade of cytokine‐mediated signaling. Notably, the combination of lusvertikimab and imatinib led to a decrease in pSTAT5 below baseline levels (P = 0.0462), with no restoration of IL‐7R‐pathway activity upon IL‐7‐stimulation. Consistently, combination treatment reduced cell viability in assays employing a higher imatinib concentration (Supporting Information S1: Figure 4A). In 2/6 tested PDX‐samples we observed IL‐7‐mediated pSTAT5 upregulation that could be blocked by lusvertikimab (Supporting Information S1: Figure 4B). In both PDX‐samples, imatinib alone effectively reduced pSTAT5, but, unlike in SUP‐B15, this suppression was not reverted by IL‐7‐exposure.

Together, these data suggest that IL‐7R/STAT5 pathway inhibition may contribute to lusvertikimab/TKI combination efficacy, whereas CD127 surface expression appears to represent a more robust and clinically accessible biomarker of lusvertikimab sensitivity.

Finally, to evaluate whether lusvertikimab can be effectively combined with ABL‐targeting TKIs in vivo, we performed additional phase‐2‐like PDX studies using PDX#1–8 in an overt leukemia setting (Figure 2C). In line with previous models,^6^ imatinib monotherapy showed only modest in vivo efficacy. Imatinib/lusvertikimab combination treatment was well tolerated and resulted in improved leukemia control, showing a significant survival benefit compared with controls (P = 0.014, Figure 2D, Supporting Information S1: Figure 5A–D). However, the additional benefit over lusvertikimab monotherapy remained heterogeneous across models.

In vitro analyses showed comparable mechanistic interactions for dasatinib/lusvertikimab and imatinib/lusvertikimab, including enhanced ADCP and cooperative inhibition of IL‐7R/STAT5 signaling (Supporting Information S1: Figure 6A–D). We therefore next evaluated dasatinib/lusvertikimab across all models in vivo. In contrast to imatinib, dasatinib monotherapy already showed marked antileukemic activity compared with controls (P = 0.0302, Figure 2E, Supporting Information S1: Figure 7A,B). Importantly, dasatinib/lusvertikimab combination demonstrated the most pronounced overall in vivo efficacy, significantly prolonging survival compared with controls (P = 0.0001) and dasatinib monotherapy (P = 0.0057). Importantly, while the additional effect over lusvertikimab monotherapy did not reach statistical significance (P = 0.1126), the dasatinib/lusvertikimab combination induced long‐term survival in the majority of tested PDX models, with 5 of 8 animals reaching the end of the 130‐day observation period. Notably, therapeutic benefit was observed across all tested PDX models, including those that had shown limited additional benefit with the imatinib/lusvertikimab combination (Figure 2E, Supporting Information S1: Figure 7A,B).

Together, these data show that lusvertikimab can be effectively combined with ABL‐targeting TKIs and that therapeutic efficacy is strongly influenced by the potency of the TKI backbone.

In *BCR::ABL1*+ B‐ALL, IL‐7‐signaling plays a particular role because it acts as an accomplice, reinforcing leukemogenic pathways activated by the fusion kinase, also in the context of TKI treatment.^6^ Here, we provide mechanistic data supporting a chemo‐free combination therapy with TKI due to multi‐level interactions.

First, as with BCR::ABL1 mouse models, TKI treatment readily increased surface CD127 expression in human cell lines and PDX‐samples, potentially allowing enhanced binding of CD127‐directed antibodies. Second, TKI treatment further enhanced lusvertikimab‐mediated ADCP in vitro, and third, TKI + lusvertikimab synergized in blocking cell‐extrinsic and ‐intrinsic IL‐7Rα‐downstream signaling via STAT5, potentially circumventing important therapy escape mechanisms previously demonstrated.^16^ Our data therefore suggest relevant baseline efficacy of lusvertikimab, even in CD127‐low cases, while combined targeting of IL‐7R signaling and ABL kinase activity provides a rationale for chemo‐free therapeutic strategies.

For our study, imatinib was initially selected as a “model TKI” based on its antileukemic activity across a variety of ABL‐class fusions and prior mechanistic evidence linking IL‐7 signaling to imatinib response in BCR::ABL1‐positive ALL.^17^ Results obtained with dasatinib, showing comparable mechanistic cooperation with lusvertikimab but superior in vivo efficacy, indicate that this therapeutic concept is not restricted to imatinib and can be extended to more potent ABL‐targeting TKIs.

Consistent with known limitations of antibody‐based approaches in the CNS, all imatinib/lusvertikimab‐treated animals in our model eventually developed CNS disease (Supporting Information S1: Figure 5D), underscoring the persisting challenge of CNS involvement in ALL. It will therefore be important to determine whether individual TKIs differ in their ability to support CD127‐directed combination therapy with regard to CNS disease control.

“Chemotherapy‐free” regimens including TKI and blinatumomab have shown promising success in ABL‐class‐fusion+ B‐ALL, but not all patients benefit, particularly high‐risk *IKZF* ^*plus*^ and CD19‐negative cases.^18^ Notably, responses to lusvertikimab/TKI combinations were also observed in a CD19‐negative r/r PDX model and in *IKZF* ^*plus*^ samples (Supporting Information S1: Table 1), highlighting the potential relevance of this strategy in clinically challenging subgroups.

Given the pronounced single‐agent activity of lusvertikimab and the improved efficacy observed in combination with potent TKIs, further studies are warranted to define the patient populations most likely to benefit from CD127‐directed combination approaches and the optimal TKI backbone in this context.

First clinical trials to test the efficacy of antibody‐based CD127‐IT in relapsed/refractory B‐ and T‐ALL patients are currently being initiated. Our data support the inclusion of ABL‐class‐fusion+ patients in such future trial protocols.

## AUTHOR CONTRIBUTIONS

**Denis Pinkle**: Investigation; writing—original draft; methodology. **Melissa Cornils**: Investigation; visualization; writing—review and editing. **Anna Dietterle**: Investigation. **Anna Laqua**: Investigation; methodology. **Michelle Kurschies**: Investigation. **Julia Heymann**: Investigation. **Emily Funke**: Investigation; data curation. **Philipp Fanter**: Investigation; data curation. **Dennis D. Gupta**: Data curation; investigation; writing—original draft. **Claas Reimer**: Investigation. **Denny Takudzwa Mahachi**: Investigation. **Carina L. Gehlert**: Investigation. **Dorothee Winterberg**: Investigation. **Michaela Vossen‐Gajcy**: Investigation; data curation. **Xenia Müller**: Investigation. **Greta Balow**: Investigation. **Katharina Iben**: Investigation. **Malwine Barz**: Investigation. **Lorenz Bastian**: Investigation; data curation. **Claudia Baldus**: Resources. **Maja Kowol**: Investigation; visualization. **Sally Kikvadze**: Investigation. **Alexander A. Wurm**: Investigation; resources. **Ekaterina Logvinova**: Investigation. **Christian Vokuhl**: Investigation. **Jan P. Schmid**: Investigation. **Irmela Jeremias**: Resources. **Katja Klausz**: Investigation. **Matthias Peipp**: Supervision; resources. **Martin Schrappe**: Resources; investigation. **Irène Baccelli**: Investigation; resources. **Nicolas Poirier**: Resources. **Monika Brüggemann**: Methodology; resources; investigation. **Gunnar Cario**: Supervision; resources; funding acquisition. **Denis M. Schewe**: Conceptualization; methodology; investigation; supervision; resources; writing—review and editing; funding acquisition. **Lennart Lenk**: Conceptualization; methodology; investigation; supervision; visualization; project administration; resources; writing—original draft; funding acquisition.

## CONFLICT OF INTEREST STATEMENT

L.L. received research funding from OSE Pharmaceuticals. M.B. received fees for speakers bureau/travel support by Amgen, BD, Janssen, and Pfizer and was an advisory board member for Amgen, AstraZeneca, Hello Healthcare, and Incyte. D.M.S. was an advisory board member for Bayer, SOBI, and Jazz Pharmaceuticals and received research funding from OSE Pharmaceuticals. G.C. received research and education funding from Amgen, Clinigen, Jazz, and Servier; travel support from Jazz; and is a board member for Amgen. M.S. received research funding from Servier, as well as fees for speaker Bureaus from Jazz and Amgen. C.B. has received consultancy fees and honoraria from Janssen, Astellas, Pfizer, AstraZeneca, Servier, and Bristol Myers Squibb. I.B. and N.P. are/were employees and shareholders of OSE Immunotherapeutics, a company owning the anti‐CD127‐antagonist OSE‐127. They are authors of patents related to CD127‐antagonist antibodies. The remaining authors have declared no conflicts of interest.

## ETHICS STATEMENT

The study was approved by the ethical committee of the Christian‐Albrecht University Kiel (D437/17). Leukemia xenografts were generated in accordance with local governmental regulations (Schleswig‐Holstein Ministerium für Landwirtschaft, ländliche Räume, Europa und Verbraucherschutz). This information is also stated in the methods section of the supplementary data. Patients included in this research project were treated according to AIEOP‐BFM ALL protocols after informed consent in accordance with the Declaration of Helsinki.

## FUNDING

LL, AD, JH, MK, and CR have been supported by the Faculty of Medicine, University of Kiel, Germany. DP has been supported by a scholarship from the GPOH and the Deutsche Jose Carreras Leukämiestiftung. LL, DMS, MB, LB, DDG, MP, GC, MS, CB, and MB are members of the Clinical Research Unit “CATCH ALL” (KFO 5010) funded by the Deutsche Forschungsgemeinschaft (DFG), Bonn, Germany. Open Access funding enabled and organized by Projekt DEAL.

## Supporting information

Supplementary Data.

## Data Availability

The data that support the findings of this study are available in the main manuscript, figures, and supplementary material of this article. All data underlying the presented results are contained in the main manuscript, figures, or supplementary data.
