# Supplementary material for: Complementary efficacy of CD127‐directed immunotherapy with lusvertikimab and ABL‐targeting tyrosine kinase inhibitors in preclinical ABL‐class‐fusion‐positive B‐ALL
Source: Hemasphere. 2026 Jun 17;10(6):e70407. doi: 10.1002/hem3.70407 (PMC13274471; doi:10.1002/hem3.70407)
Supplement: Supplementary file 1 — Supplementary Data. [file HEM3-10-e70407-s001.docx]

| PDX # | Age Group | Age at diagnosis (years) | Sex | WBC | Relapse | Death | Immunophenotype | Cytogenetics^1^ | *IKZF*  status^2^ | Sample origin | Delta PBB LUSV^3^ | %CD127+ cells^4^ | %CD127+ cells Imatinib^4^ |
| --- | --- | --- | --- | --- | --- | --- | --- | --- | --- | --- | --- | --- | --- |
| PDX1 | Pediatric | 17.3 | F | 13300 | Yes | No | common B-ALL | *BCR::ABL1* | Wildtype | De novo B-ALL | 72.21 | 54.8 | 62.9 |
| PDX2 | Pediatric | 9.8 | F | 132000 | No | No | common B-ALL | *BCR::ABL1* | *IKZF^plus^* | De novo B-ALL | 62 | 63 | 88.8 |
| PDX3 | Pediatric | 9 | M | 3500 | N/A | N/A | N/A | *BCR::ABL1* | *IKZF^plus^* | De novo B-ALL | 84 | 74.4 | 75.8 |
| PDX4 | Adult | 24 | F | 124000 | No | No | Common B-ALL | *BCR::ABL1* | *IKZF^plus^* | De novo B-ALL | 68 | 74.6 | N/A |
| PDX5 | Adult | 89 | F | 750 | No | No | common B-ALL | *BCR::ABL1* | Wild  type | De novo B-ALL | 72.26 | 80.7 | 91.7 |
| PDX6 | Pediatric | 1.6 | M | 288400 | No | No | common B-ALL | *EBF1::PDGFRβ* | *IKZF^plus^* | De novo B-ALL | 36.7 | 45.9 | 78.6 |
| PDX7* | Young Adult | 19 | M | 11500 | Yes | Yes | common B-ALL | *ETV6::ABL1* | *IKZF^plus^* | R/R B-ALL | 57.5 | 85.1 | 87.2 |
| PDX8* | Young Adult | 19 | M | 11500 | Yes | Yes | common B-ALL | *ETV6::ABL1* | *IKZF^plus^* | R/R B-ALL | 45.6 | 82.7 | 92.1 |

**Supplementary Table 1: Clinical characteristics of B-ALL PDX samples used in phase-2-like overt leukemia study and *in vitro* analysis (refers to Figure 1 and Figure 2)**
^1^ Stratification relevant lesions were diagnosed by fluorescence in situ hybridization or targeted RNA sequencing as described previously ^1^.

^2^ *IKZF^plus^* was defined as described in Stanulla *et al*, JCO, 2018)*^2^. IKZF^plus^*-relevant genes were analyzed via multiplex ligation-dependent probe amplification (MLPA) in PDX cells.

^3^ Reduction of peripheral blood blasts (PBB) of LUSV-treated mice compared with control-treated mice by the time one of the corresponding PDX animals or animal groups showed signs of overt leukemia (ΔPBB = PBB_Control-treated_ – PBB_LUSV-treated_).

^4^ CD127+ cells of all cells in the ALL cell population as determined via flow cytometry.

^5^ CD127+ cells of all cells in the ALL cell population as determined via flow cytometry after 48h of *in vitro* treatment with 1µM imatinib.

* PDX sample generated from same donor. PDX8 was depleted of CD19 by CRISPR/Cas9 editing, PDX7 is the corresponding wildtype sample. PDX8 was not included into statistical survival assessments of *in vivo* phase-2-like PDX trials.

**Supplementary Figures**


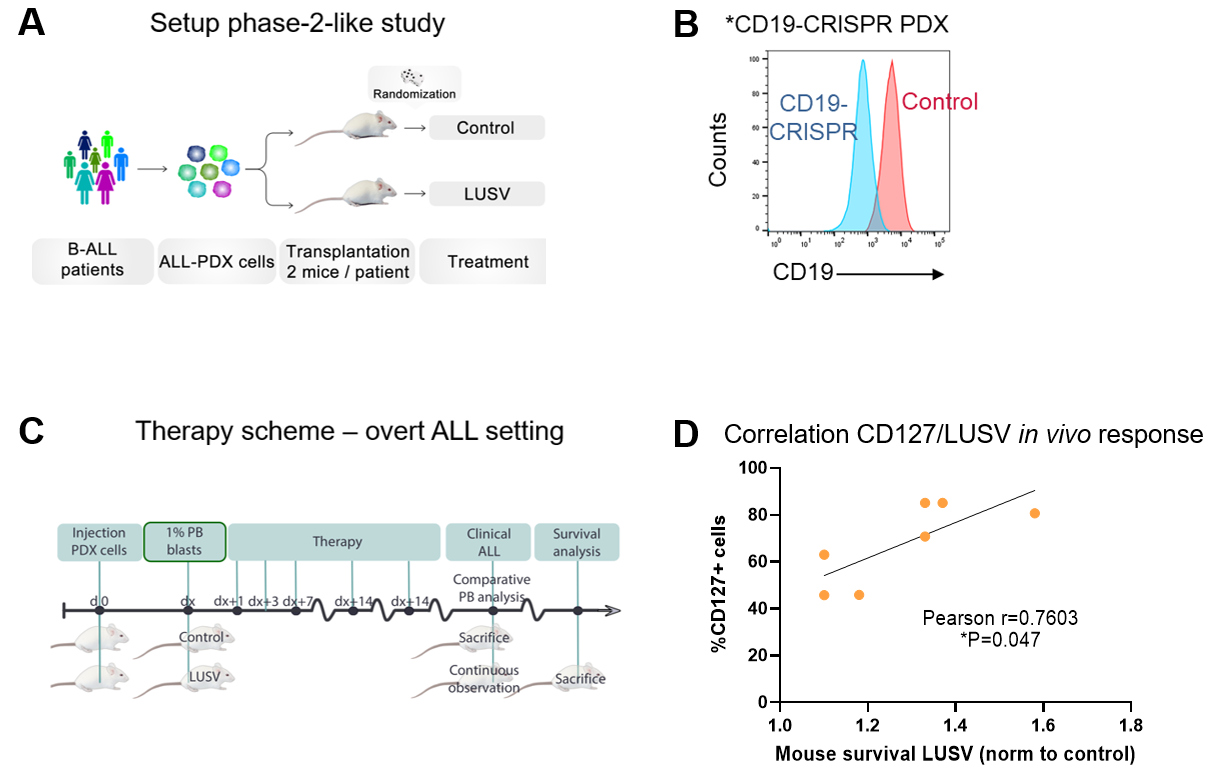


**Supplementary Figure 1 - Lusvertikimab (LUSV) is preclinically active in ABL-class-fusion-positive ALL: A)** Experimental setup of a phase-2-like PDX trial to test the in vivo efficacy of LUSV as compared to control. **B)** The knockout of CD19 was performed as described previously ^3^. The knockout efficiency was validated with flow cytometry analysis within the CD45-positive population and using fluorescence-minus-one (FMO) controls. **C)** Treatment scheme of phase-2-like PDX trial. **D)** Correlation analysis of CD127+ cells in PDX cells versus survival of LUSV-treated mice normalized to control.


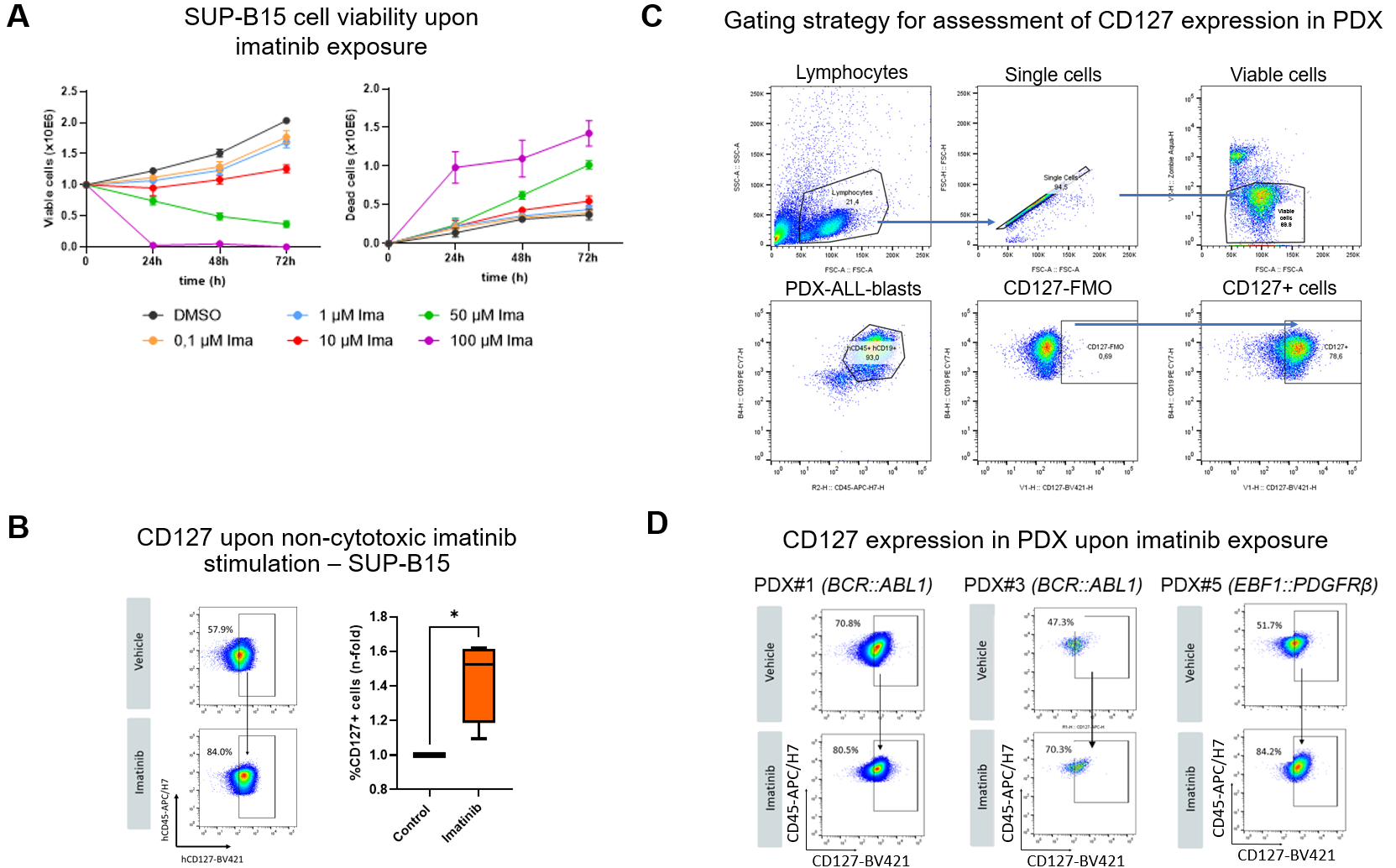


**Supplementary Figure 2 - CD127 is targetable in ABL-class-fusion+ ALL and its expression can be enhanced by imatinib treatment:** SUP-B15 cells were treated with different concentrations of imatinib (Ima) and cell viability was measured via trypan-blue exclusion assay after 24h, 48h, and 72h. 1µM was chosen as sublethal working concentration for further experiments. B-D) SUP-B15 cells or ABL-class-fusion+ PDX cells were exposed to sublethal doses of imatinib (1µM) or DMSO control and subjected to CD127-assessment via flow cytometry after 48h. B) Representative flow cytometry images and two-tailed Mann-Whitney test of n=4 experiments with SUP-B15 cells. **C)** Gating strategy to assess blast frequency and CD127 levels in PDX cells. **D)** Representative images of CD127 flow cytometry analyses before and after 48h imatinib treatment.


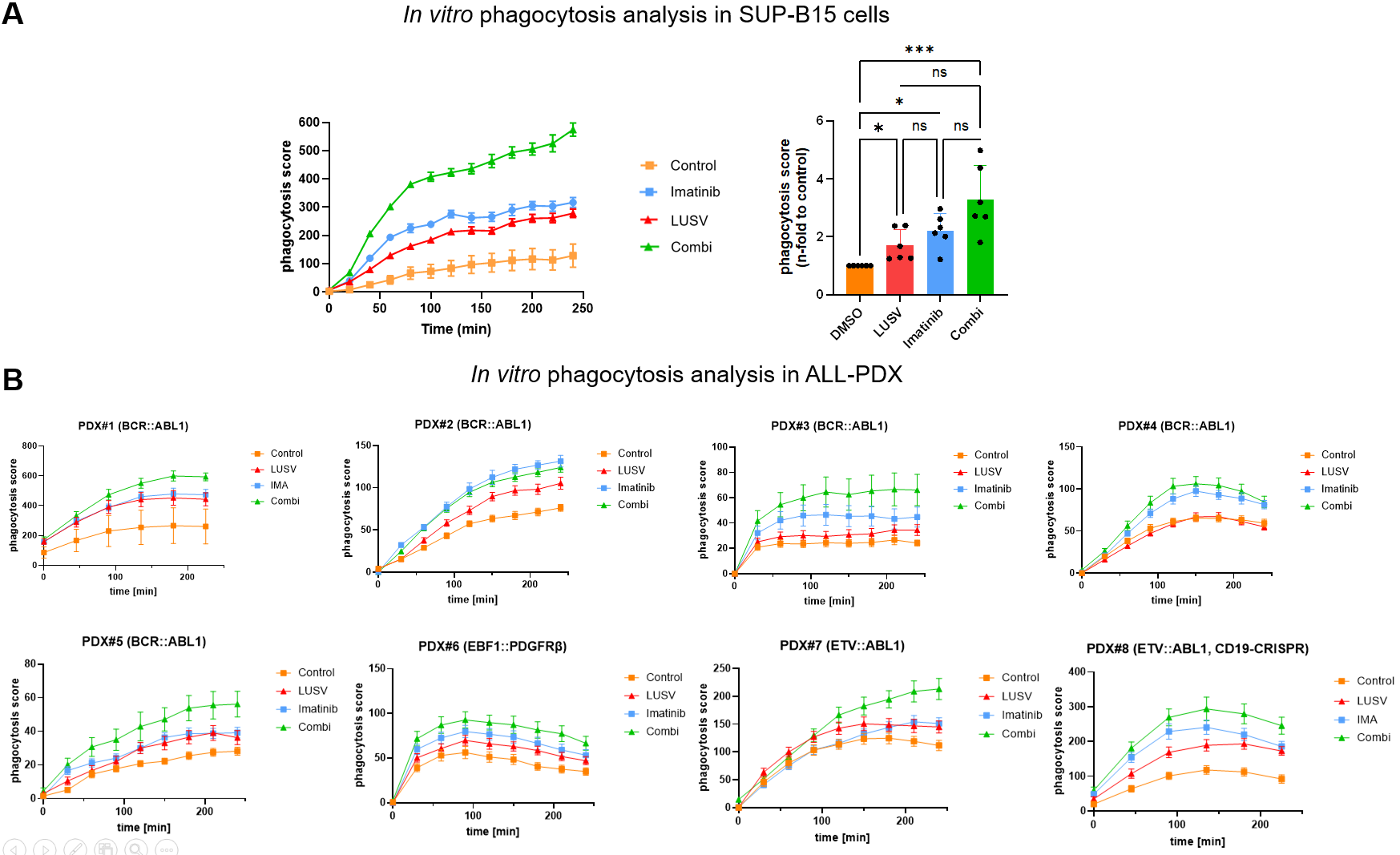


**Supplementary Figure 3: Imatinib enhances LUSV-mediated antibody-dependent cellular phagocytosis (ADCP) by macrophages in ABL-class-fusion+ ALL cells: A)** BCR::ABL1+ SUP-B15 cells were treated with sublethal doses of imatinib (Ima, 1µM) for 48h versus DMSO control and analyzed for phagocytosis/ADCP using primary macrophages from healthy donors as effector cells and IncuCyte live-cell imaging as readout. Phagocytosis levels of a representative experiment and one-way ANOVA of n=6 experiments are shown. **B)** ABL-class-fusion+ ALL cells were treated with sublethal doses of imatinib (1µM) for 48h versus DMSO control and analyzed for phagocytosis/ADCP using primary macrophages from healthy donors as effector cells and IncuCyte live-cell imaging as readout. All samples from our PDX cohort (PDX1-8) were analyzed.


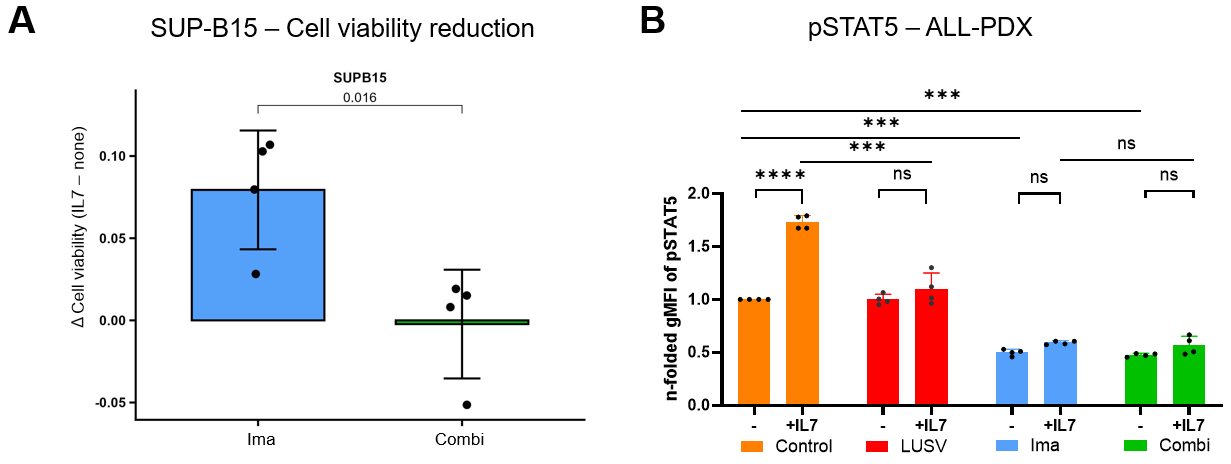


**Supplementary Figure 4:** **Imatinib enhances LUSV-mediated STAT5-blockade in ABL-class-fusion+ ALL cells:A)** SUP-B15 cells were pre-incubated with LUSV (1 µg/mL) or control and cultured in the presence of a cytotoxic imatinib dose (12.5µM) with or without recombinant human IL-7 and the reduction of cell viability (delta cell viability) was analyzed via propidium iodide exclusion, two-tailed t-test. **B)** The effect of STAT5-phosphorylation (pSTAT5) in ABL-class-fusion+ PDX cells treated with non-cytotoxic Ima and/or LUSV in the presence or absence of recombinant IL-7 was analyzed. Analysis of two IL-7-responsive PDX samples are shown. P < .05; ∗∗P < .01; ∗∗∗P < .001; ∗∗∗∗P < .0001


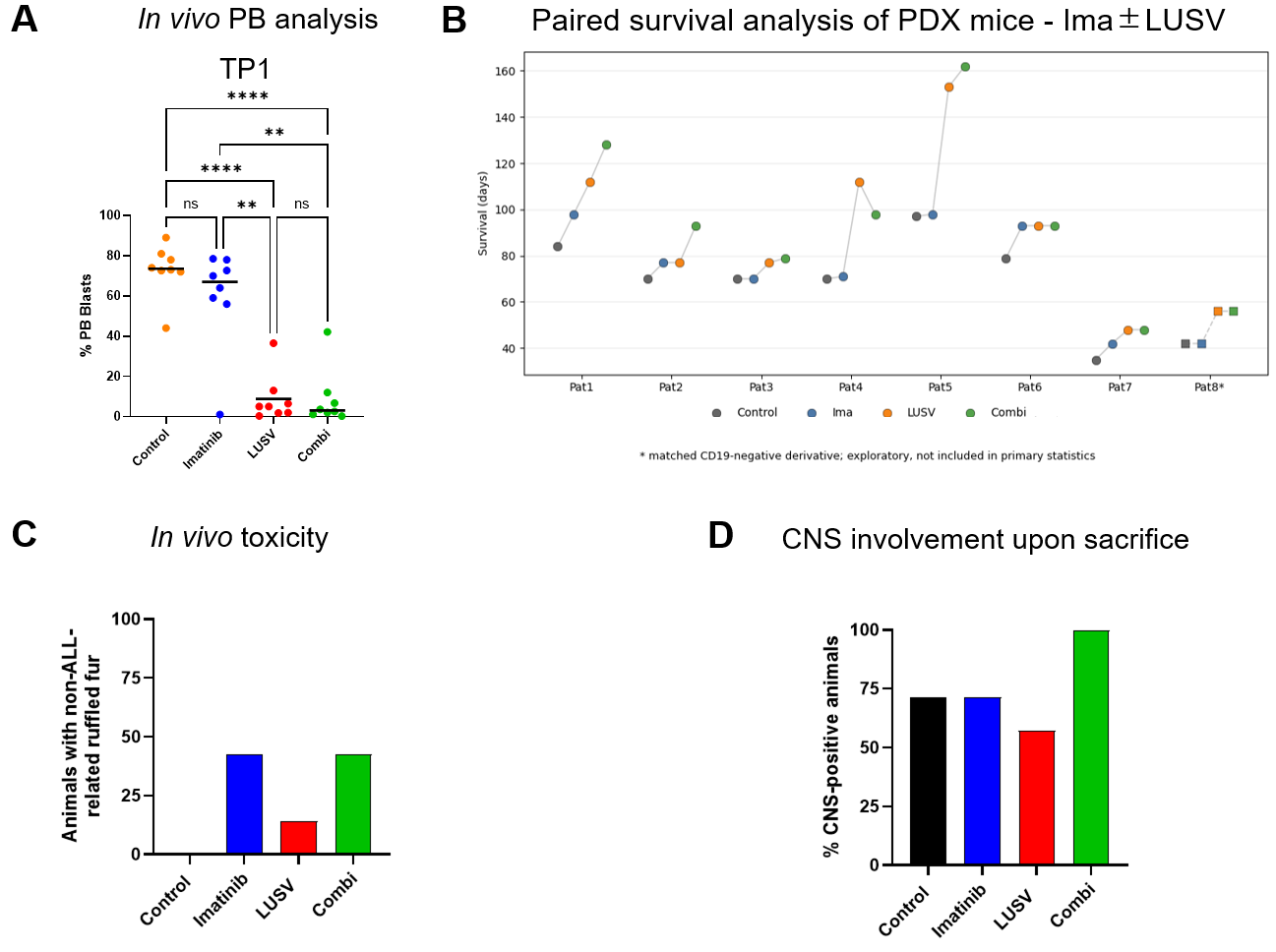


**Supplementary Figure 5: Imatinib plus Lusvertikimab (LUSV) can be combined to efficiently target ABL-class-fusion+ ALL cells in vivo:** A phase-2-like PDX study was performed using 8 PDX samples of different ABL-class-fusion+ patients (7 independent patient-derived PDX samples and CD19-edited derivative of PDX#7). Four NSG mice per patient were injected with PDX cells, randomly assigned into treatment groups, and LUSV and/or imatinib therapy was initiated upon detection of 1% PDX cells in the PB, modeling an overt leukemia situation. **A)** Blood of all control and LUSV/Ima-treated animals bearing the same PDX sample was withdrawn when 1 of the 4 PDX mice showed clinical signs of overt leukemia, and the number of hCD45+/hCD19+/mCD45– cells in the peripheral blood was measured via flow cytometry, **B)** Paired survival analysis of all 8 PDX models. **C)** Animals were checked daily for leukemia and toxicity-associated signs. Ruffled fur in animals without clinical overt leukemia was assessed as a sign of treatment-related toxicity. **D)** After sacrifice, animals were checked for organ-specific engraftment in the spleen, bone marrow, and CNS. For the analysis of CNS-infiltration, head sections were stained with hematoxylin/eosin and assessed for the presence of blast in a semi-quantitative manner as described previously in a blinded fashion by a pathologist ^4–6^. P < .05; ∗∗P < .01; ∗∗∗P < .001; ∗∗∗∗P < .0001


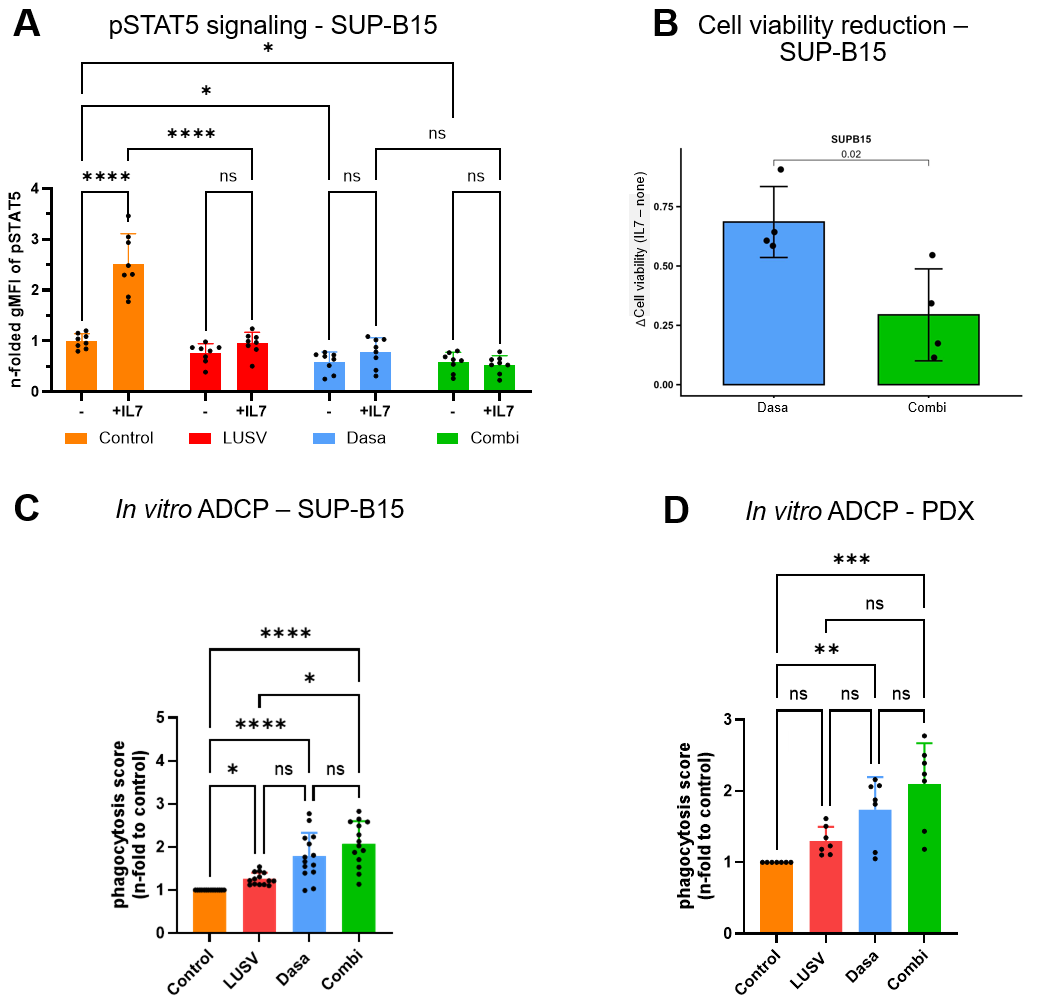


**Supplementary Figure 6:** **Dasatinib enhances LUSV-mediated STAT5-blockade and macrophage-mediated phagocytosis (ADCP) of ABL-class-fusion+ ALL cells:**

**A)** The effect of STAT5-phosphorylation (pSTAT5) in *BCR::ABL1*+ SUP-B15 cells treated for 1 hour with 1µM dasatinib (Dasa) and/or LUSV in the presence or absence of recombinant IL-7 was analyzed. **B**) BCR::ABL1+ SUP-B15 cells were treated with sub-cytotoxic doses of dasatinib (Dasa, 10nM) for 48h versus DMSO control and analyzed for STAT5-phosphorylation in the presence or absence of recombinant IL-7, analysis of n=4 individual experiments are shown; two-way ANOVA. **B**) SUP-B15 cells were pre-incubated with LUSV (1 µg/mL) or control and cultured in the presence of increasing dasatinib concentrations with or without recombinant human IL-7 and the reduction of cell viability (delta cell viability) was analyzed via propidium iodide exclusion, two-tailed t-test. **C-D)** ABL-class-fusion-positive cells were treated with sublethal doses of dasatinib (Dasa, 10nM) for 48h versus DMSO control and analyzed for phagocytosis/ADCP upon LUSV or control treatment using primary macrophages from healthy donors as effector cells and IncuCyte live-cell imaging as readout. One-way ANOVA of **C)** n=14 experiments with SUP-B15 cells and **D)** n=7 individual PDX samples are shown. P < .05; ∗∗P < .01; ∗∗∗P < .001; ∗∗∗∗P < .0001


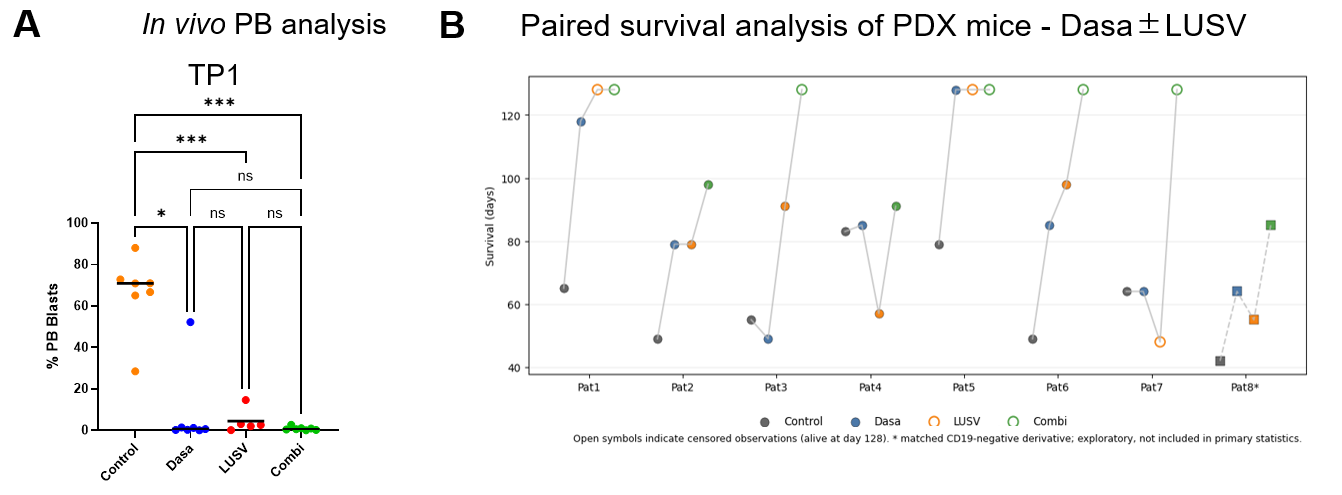


**Supplementary Figure 7: Dasatinib (Dasa) plus Lusvertikimab (LUSV) efficiently target ABL-class-fusion+ ALL cells in vivo:** A phase-2-like PDX study was performed using 8 PDX samples of different ABL-class-fusion+ patients. Four NSG mice per patient were injected with PDX cells, randomly assigned into treatment groups, and LUSV and/or dasatinib therapy was initiated upon detection of 1% PDX cells in the PB, modeling an overt leukemia situation. **A)** Blood of all control and LUSV/Dasa-treated animals bearing the same PDX sample was withdrawn when 1 of the 4 PDX mice showed clinical signs of overt leukemia, and the number of hCD45+/hCD19+/mCD45– cells in the peripheral blood was measured via flow cytometry, **B)** Paired survival analysis of all 8 PDX models. 5/8 dasatinib/Lusvertikimab-treated mice survived the observation time of 130 days and were marked as empty green points (PDX#1, #3, #5, #6, #7). One mouse died due procedural reasons and was censored as empty orange point (PDX#7-LUSV). < .05; ∗∗P < .01; ∗∗∗P < .001; ∗∗∗∗P < .0001

**Supplementary Methods:**

**Patients -** Patients with leukemia were treated according to AIEOP-BFM ALL 2000 or 2009 protocols, COALL or GMALL study group protocols after informed consent in accordance with the Declaration of Helsinki. Prospective measurements of CD127 were conducted in patients included in the AIEOP-BFM ALL 2017 or EsPhALL 2017 study after informed consent. The study was approved by the ethical committee of the Christian-Albrecht University Kiel (D437/17).

**Flow cytometry -** Prospective measurements of diagnostic ALL patient samples were conducted in accordance with EuroFlow standards and gated as described previously ^7–9^.

For flow cytometry analyses of PDX cells, at least 1x10^6^ PDX cells (ficollized, cryopreserved or fresh) were washed with PBS and subjected to fixable viability stainings using the Zombie Aqua Fixable Viability Kit (Biolegend). Cells were washed in FACS-Buffer and incubated with primary antibody cocktail for 30 min at 8°C for surface staining. The following antibodies were used for flow cytometry:

| Target | Structure | Origin | Clone | Dye | Reference | Company |
| --- | --- | --- | --- | --- | --- | --- |
| hCD127 | surface | mouse | A019D5 | APC | 351316 | Biolegend |
| hCD127 | surface | mouse | HIL-7R-M21 | BV421 | 557938 | BD Biosciences |
| hCD19 | surface | mouse | HIB19 | PE/Cy7 | 302216 | Biolegend |
| hCD19 | surface | mouse | HIB19 | PE | 302208 | Biolegend |
| hCD45 | surface | mouse | 2D1 | APC/H7 | 560274 | BD Pharmigen |
| hCD45 | surface | mouse | HI30 | FITC | 304006 | Biolegend |
| mCD45 | surface | rat | 30-F11 | APC | 17-0451-82 | Invitrogen |

Samples were directly analyzed using a MACSQuant X (Miltenyi Biotec) flow cytometer. A total of 100,000 events were collected from each sample when possible. The lymphocyte gate was analyzed depending on distinguished FSC vs. SSC properties. Singlets were then selected (FSC-A vs FSC-H). The living cells were identified using Zombie Aqua Fixable Viability dye and further analyzed according to their surface or intracellular protein stains. B-ALL-cells were identified by hCD19+/hCD45dim, normal B-cells by hCD19+/hCD45high and T-cells by hCD7+/hCD45high staining features. To indicate the boundaries between sCD127 negative and positive populations in B-ALL and T-ALL samples, gates were set according to a fluorescence-minus-one (FMO) control-stained probe (Supplementary Figure 1A). FlowJo v.10.7 was used for data analysis.

**Antibodies -** Therapeutic anti-CD127 antibodies were generated by OSE Immunotherapeutics and found free from endotoxin.

**Xenograft models -** Leukemia-xenografts were generated in accordance with local governmental regulations (Schleswig-Holstein Ministerium für Landwirtschaft, ländliche Räume, Europa und Verbraucherschutz). NOD.Cg-Prkdcscid Il2rgtm1Wjl/SzJ (NSG)-mice were purchased by Charles River (Sulzfeld, GER). PDX-cells were injected intravenously into female NSG-mice (6–10 weeks of age). For overt leukemia experiments, mice were injected with 1x10^6^ ALL-PDX-cells and therapy was started upon detection of 1% PDX-ALL cells in the peripheral blood (PB). Imatinib (40 mg/kg) and dasatinib (10 mg/kg) were administered via daily oral gavage as described previously ^10^.

Animals were sacrificed when showing signs of overt leukemia (detection of >70% leukemic blasts in the PB or clinical signs of leukemia including loss of weight or activity, organomegaly, hindlimb paralysis ^4–6,11–14^.

**Cell lines –** The human cell line SUP-B15 was purchased from DSMZ (Leibniz Institute, Germany). All cells were tested and found free from mycoplasma. Cells were regularly authenticated via short-tandem repeat (STR) analysis.

**Phagocytosis assays –** ABL-class-fusion+ ALL cell lines and PDX cells were treated with TKI (1µM imatinib or 10nM dasatinib) or DMSO control, and *in vitro* phagocytosis was analyzed as described previously ^9,11,15^.

For this analysis, macrophages were generated from healthy human donor blood samples as previously described ^16^. Then, 2×10^4^ macrophages were plated on a 96-well plate and allowed to adhere at room temperature for 30 minutes. Next, 1x10^6^ target cells were stained with 0.5 µg/mL pHrodo (Thermo Fisher Scientific) for 1 hour at room temperature. LUSV was applied at a final concentration of 0.1 µg/mL and imatinib at 1µM. *In vitro* phagocytosis was measured by using IncuCyte live-cell imaging. Engulfed cells are displayed as red object counts per image (phagocytosis score).

**Phosphorylated (P)-STAT5 probing by flow cytometry -** Cells were seeded in 96 well plates at 3x10^4^ / 100 µl medium and treated with the TKIs imatinib (1µM) or dasatinib (1nM), LUSV (1µg/mL), the combination of LUSV and TKI or just medium (control). LUSV was added 2 hours prior to other indicated treatments to ensure receptor blockage. Where indicated, IL7 was added in a final concentration of 0.01 µg/µL. Cells were harvested, washed with 2% FCS/PBS), and stained with Zombie Violet Viability Dye (BioLegend) diluted 1:500 in PBS for 15 minutes at room temperature in the dark. After washing, cells were fixed with 4% paraformaldehyde for 10 minutes at 37°C, washed, and permeabilized by incubation in ice-cold 100% methanol for 30 minutes. For long-term storage, cells were permeabilized in 90% methanol / 10% PBS and stored at −20°C. Cells were then rehydrated and washed with Phosflow wash buffer (PBS + 2% FCS + 0.2% Tween-20). Intracellular staining was performed in individual tubes using PE-conjugated anti-phospho-STAT5 antibody (RRID: AB 10980279) in Phosflow buffer for 1 hour at room temperature in the dark.

**TKI/LUSV cell viability reduction analysis:** SUP-B15 cells were seeded at 6×10⁴ cells per well in 96-well plates in complete McCoy medium. Cells were pre-incubated with lusvertikimab (1 µg/mL) or control medium for 1 h at 37 °C and cultured in the presence or absence of recombinant human IL-7. Cells were then treated either with a row-wise dasatinib concentration gradient (10 µM–10⁻⁶ µM) or with a fixed imatinib concentration (12.5 µM) for 48 h at 37 °C and 5% CO₂. Cell viability was assessed by propidium iodide exclusion using flow cytometry. All conditions were analyzed in duplicate measurements.

References

1. Antić Ž, van Bömmel A, Riege K, et al. Recurrent DNMT3B rearrangements are associated with unfavorable outcome in dicentric (9;20)-positive pediatric BCP-ALL. *Leukemia*. 2023;37(12):2522-2525. Published October 16, 2023.

2. Stanulla M, Dagdan E, Zaliova M, et al. IKZF1plus Defines a New Minimal Residual Disease-Dependent Very-Poor Prognostic Profile in Pediatric B-Cell Precursor Acute Lymphoblastic Leukemia. *Journal of Clinical Oncology*. 2018;36(12):1240-1249. Published March 2, 2018.

3. Bahrami E, Schmid JP, Jurinovic V, et al. Combined proteomics and CRISPR‒Cas9 screens in PDX identify ADAM10 as essential for leukemia in vivo. *Molecular cancer*. 2023;22(1):107. Published July 8, 2023.

4. Alsadeq A, Lenk L, Vadakumchery A, et al. IL7R is associated with CNS infiltration and relapse in pediatric B-cell precursor acute lymphoblastic leukemia. *Blood*. 2018;132(15):1614-1617. Published August 28, 2018.

5. Lenk L, Carlet M, Vogiatzi F, et al. CD79a promotes CNS-infiltration and leukemia engraftment in pediatric B-cell precursor acute lymphoblastic leukemia. *Communications Biology*. 2021;4(1):73. Published January 15, 2021.

6. Lenk L, Winterberg D, Vogiatzi F, et al. Preclinical Evidence for the Efficacy of CD79b Immunotherapy in B-cell Precursor Acute Lymphoblastic Leukemia. *HemaSphere*. 2022;6(8):e754. Published July 15, 2022.

7. Kalina T, Flores-Montero J, van der Velden VHJ, et al. EuroFlow standardization of flow cytometer instrument settings and immunophenotyping protocols. *Leukemia*. 2012;26(9):1986-2010.

8. Dworzak MN, Buldini B, Gaipa G, et al. AIEOP-BFM consensus guidelines 2016 for flow cytometric immunophenotyping of Pediatric acute lymphoblastic leukemia. *Cytometry Part B: Clinical Cytometry*. 2018;94(1):82-93. Published February 21, 2017.

9. Lenk L, Baccelli I, Laqua A, et al. The IL-7R antagonist lusvertikimab reduces leukemic burden in xenograft ALL via antibody-dependent cellular phagocytosis. *Blood*. 2024;143(26):2735-2748. https://pubmed.ncbi.nlm.nih.gov/38518105/.

10. Abdelrasoul H, Vadakumchery A, Werner M, et al. Synergism between IL7R and CXCR4 drives BCR-ABL induced transformation in Philadelphia chromosome-positive acute lymphoblastic leukemia. *Nature Communications*. 2020;11(1):3194. Published June 24, 2020.

11. Müller K, Vogiatzi F, Winterberg D, et al. Combining daratumumab with CD47 blockade prolongs survival in preclinical models of pediatric T-ALL. *Blood*. 2022;140(1):45-57.

12. Schewe DM, Lenk L, Vogiatzi F, et al. Larotrectinib in TRK fusion-positive pediatric B-cell acute lymphoblastic leukemia. *Blood Advances*. 2019;3(22):3499-3502.

13. Vogiatzi F, Winterberg D, Lenk L, et al. Daratumumab eradicates minimal residual disease in a preclinical model of pediatric T-cell acute lymphoblastic leukemia. *Blood*. 2019;134(8):713-716. Published July 16, 2019.

14. Winterberg D, Lenk L, Oßwald M, et al. Engineering of CD19 Antibodies: A CD19-TRAIL Fusion Construct Specifically Induces Apoptosis in B-Cell Precursor Acute Lymphoblastic Leukemia (BCP-ALL) Cells In Vivo. *Journal of clinical medicine*. 2021;10(12). Published June 15, 2021.

15. Schewe DM, Vogiatzi F, Münnich IA, et al. Enhanced potency of immunotherapy against B-cell precursor acute lymphoblastic leukemia by combination of an Fc-engineered CD19 antibody and CD47 blockade. *HemaSphere*. 2024;8(2):e48. Published February 22, 2024.

16. Schewe DM, Alsadeq A, Sattler C, et al. An Fc-engineered CD19 antibody eradicates MRD in patient-derived MLL-rearranged acute lymphoblastic leukemia xenografts. *Blood*. 2017;130(13):1543-1552. Published July 11, 2017.
